# Rare forms of small intestinal diverticulum and diagnostic features. A clinical case report

**DOI:** 10.3389/fsurg.2026.1904895

**Published:** 2026-08-19

**Authors:** Vitaliy Feoktistov, Madina Rashova, Talgat Temirbekov

**Affiliations:** 1 Department of Surgical Diseases, Karaganda Medical University, Karaganda, Kazakhstan; 2 Department of Biomedicine, Karaganda Medical University, Karaganda, Kazakhstan

**Keywords:** acute abdomen, diagnosis, emergency surgery, intestinal obstruction, jejunal diverticulitis, laparotomy, small intestinal diverticulosis

## Abstract

**Introduction:**

Small intestinal diverticulosis is a rare pathology compared with colonic diverticular disease and is often characterized by nonspecific clinical manifestations. Diagnostic difficulties increase significantly in emergency surgery, especially when diverticulitis mimics other acute abdominal conditions.

**Objective:**

To present a clinical case of jejunal diverticulosis complicated by diverticulitis and intestinal paresis, highlighting the diagnostic challenges encountered in emergency surgical practice.

**Materials and methods:**

A retrospective analysis of the clinical course, diagnostic findings, surgical treatment, and outcomes of a 69-year-old patient admitted with a presumed diagnosis of perforated gastric ulcer was performed.

**Results:**

The patient was hospitalized with acute abdominal pain, nausea, vomiting, and signs of peritoneal irritation. Laboratory examination revealed moderate leukocytosis. Plain abdominal radiography demonstrated dilated small bowel loops. Emergency laparotomy revealed serohemorrhagic peritoneal effusion, signs of acute pancreatitis, and multiple diverticula along the mesenteric border of the jejunum extending approximately 1 m from the ligament of Treitz. No evidence of perforation was found. The final diagnosis was jejunal diverticulosis complicated by diverticulitis and paralytic ileus. Conservative postoperative management, including antibacterial, detoxification, antiulcer, and symptomatic therapy, resulted in complete recovery. The patient was discharged in satisfactory condition on postoperative day 10.

**Conclusion:**

Jejunal diverticulosis may present as acute abdominal pathology and mimic intestinal obstruction or perforated peptic ulcer. Accurate diagnosis remains challenging due to nonspecific clinical and instrumental findings. In emergency settings, surgical exploration remains the most reliable diagnostic method, allowing appropriate assessment of disease severity and selection of optimal treatment strategy. The presence of small intestinal diverticulosis without perforation or severe complications does not necessarily require bowel resection.

## Introduction

Diverticular disease is one of the most common pathologies of the gastrointestinal tract, however, diverticula of the small intestine are much less common than lesions of the colon. The frequency of diverticulosis of the small intestine, according to various studies, does not exceed 0.06%–5%, while the jejunum is most often affected. The disease is mainly diagnosed in elderly patients and often remains asymptomatic for a long time (1, 2). It is estimated that small bowel diverticula account for only approximately 1%–3% of all gastrointestinal diverticula and are frequently detected incidentally during radiological examinations, endoscopy, surgery, or autopsy. The incidence increases with age, reflecting progressive degenerative changes in the intestinal wall and alterations in gastrointestinal motility (3).

Small bowel diverticula are predominantly acquired pseudodiverticula that arise due to herniation of the mucosal and submucosal layers through weak points of the muscular wall where mesenteric vessels penetrate. Increased intraluminal pressure, disorders of intestinal motility, and age-related degeneration of the smooth muscle and myenteric plexus are considered the main pathogenetic mechanisms responsible for their development (1, 4).

Despite the rarity of the pathology, the clinical significance of small intestine diverticula is due to the high risk of complications, among which diverticulitis, intestinal obstruction, bleeding, perforation and intra-abdominal abscesses are the most common. In some cases, these complications pose an immediate threat to the patient's life and require emergency surgical intervention (5–7). Approximately 10%–30% of patients eventually develop symptomatic disease or serious complications requiring hospitalization and surgical treatment, particularly elderly individuals with multiple comorbidities (6–8), at the same time, the management of such patients is complicated by the common problems of microbial resistance and the formation of biofilms in purulent-inflammatory processes in surgical practice (9).

A particular problem is the diagnosis of diverticulosis and diverticulitis of the small intestine. The clinical manifestations of the disease are nonspecific and often mimic other acute surgical diseases of the abdominal cavity, including acute pancreatitis, perforated gastric ulcer, acute appendicitis and intestinal obstruction. As a result, the correct diagnosis before surgery is established only in a limited number of patients (10). Consequently, many patients undergo emergency surgical exploration for presumed acute abdomen, and the final diagnosis is often established intraoperatively (11).

Modern methods of radiation diagnostics, primarily computed tomography of the abdominal organs, play a key role in detecting diverticula of the small intestine and their complications (10, 12). Nevertheless, even the use of high-tech research methods does not always allow timely diagnosis, especially when diverticulosis is combined with other diseases of the abdominal cavity (13). Contrast-enhanced computed tomography is currently considered the imaging modality of choice because it enables the assessment of bowel wall thickening, peridiverticular inflammatory infiltration, localized perforation, abscess formation, and signs of intestinal obstruction (11).

In recent years, there has been an increase in the number of publications devoted to complicated forms of small bowel diverticulosis, however, most of the works are represented by individual clinical observations, which indicates the continuing lack of knowledge of this problem (5, 12, 13). The rarity of the disease precludes the development of evidence-based clinical guidelines, and current therapeutic strategies are largely based on case reports, small case series, and expert opinion (1).

Due to the rarity of the disease, the difficulties of preoperative diagnosis and the lack of unified approaches to the choice of therapeutic tactics, clinical observations of patients with complicated forms of small bowel diverticulosis are of interest. The purpose of this work is to demonstrate a rare clinical case of multiple jejunal diverticulosis complicated by diverticulitis and intestinal paresis, as well as to analyze the features of its diagnosis and treatment.

## The clinical case

Patient S., 69 years old, was urgently admitted to the hospital on 29.10.25 at 21:30 with a diagnosis of a covered perforated gastric ulcer.

Upon admission, he complained of abdominal pain, nausea, vomiting, dry mouth, and weakness.

He was ill for 2 days, when sharp epigastric pain first appeared, nausea increased in pain dynamics, called an ambulance, was taken to the hospital, examined by a surgeon, hospitalized. From anamnesis: Botkin, tuberculosis, sexually transmitted diseases—denies. In 2010, he suffered a compression fracture of the cervical and lumbar spine. Disabled person of the 2nd group. Previously, he was registered with a therapist for a stomach ulcer. I did not receive anti-ulcer therapy. In 1993, he underwent an appendectomy.

### Objectively

Auscultation is vesicular respiration, it is carried out in all pulmonary fields, there is no wheezing. The heart rate is 20 per minute. The heart tones are muted, rhythmic. Arterial pressure is 100 and 70 mmHg. pulse of satisfactory properties is 100 beats per minute. The tongue is dry, covered with a brown coating. The abdomen is swollen, more in the upper parts, participates in the act of breathing. There is a 7.0 × 1.0 cm scar in the right iliac region with no signs of inflammation. On palpation, it is tense in the upper parts, painful in all parts. The Shchetkin-Blumberg symptom is positive in the upper regions.

### The results of additional research methods

Blood test: hemoglobin—156 g/L, erythrocytes—5.24 × 10^12^/L, leukocytes—12.9 × 10^9^/L, erythrocyte sedimentation rate—6 mm/h. Biochemical analysis: Blood bilirubin: total 16.8 mmol/L, direct—12.7; indirect—4.1 mmol/L; alanine transaminase—29 U/L, aspartate aminotransferase—30 U/L. Urea 4.0 mmol/L, creatinine 78 mmol/L, total protein 74 g/L, potassium 3.89 nmol/L, sodium 141.8 mmol/L, total amylase 36 mmol/L.

An overview x-ray of the abdominal cavity—small intestinal arches are detected in the middle floor of the abdominal cavity.

The patient was admitted with a suspected perforated stomach ulcer. After the preoperative preparation, the patient was taken for surgery, operated on for vital reasons on an emergency basis.

## Treatment

29.10.25—laparotomy was performed. Revision of the abdominal cavity, drainage of the abdominal cavity.

**Figure 1:**
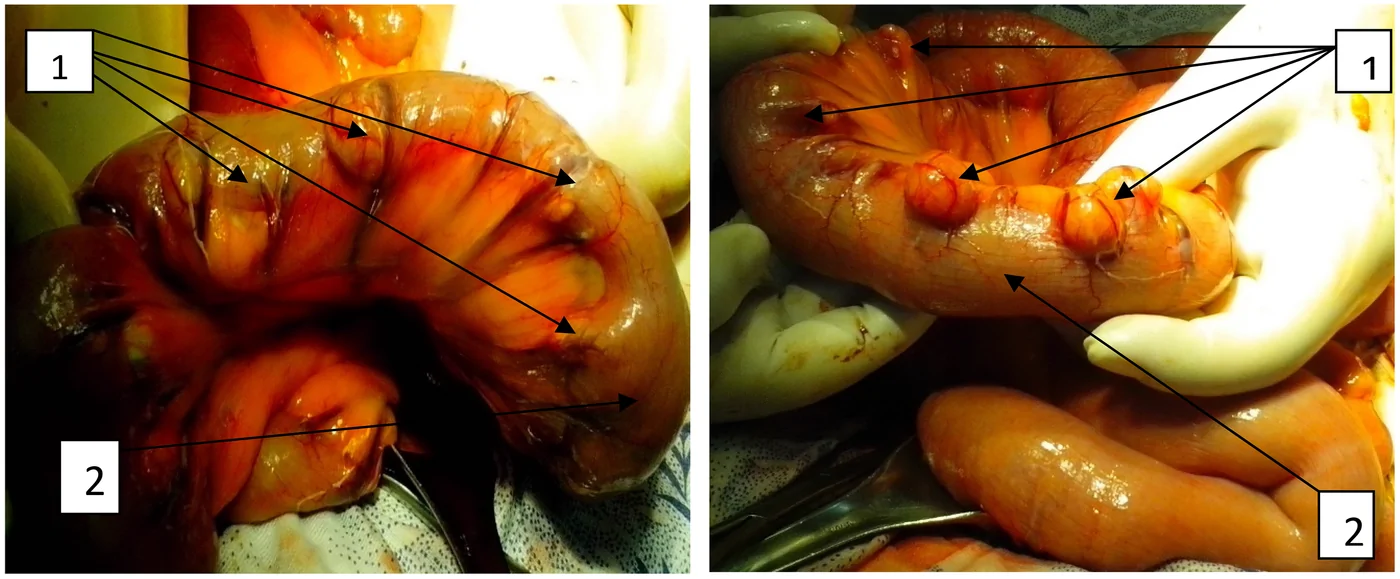
(1) diverticula, (2) jejunum.

An upper-median laparotomy was performed under endotracheal anesthesia. During the revision of the abdominal cavity, a scanty effusion of a serous—hemorrhagic nature along the right lateral canal and in the right hypochondrium. There is a pronounced adhesive process in the right subhepatic space, which involves the gallbladder, omentum, duodenum and hepatoduodenal ligament. Since the adhesive process is stale, they did not separate it. Upon examination of the stomach and duodenum, no defects were detected, upon opening the omentum, the latter was sealed, almost obliterated, the pancreas was thickened and compacted in the area of the head, elastic in the area of the body and tail, and the lobule was preserved. This condition is classified as acute pancreatitis. Upon further revision, it was found that the entire small intestine is uniformly swollen, hyperemic, and paretic. Along the mesenteric edge of the jejunum, 30 cm from the ligament of the Tracer and up to 1 m long, there are multiple diverticula with a diameter of 0.5 to 2.0 cm without signs of perforation (Figure 1). The pathology found is regarded as paresis of the small intestine, jejunum diverticulitis. Further revision revealed no other pathology. The contents of the small intestine are expressed through a nasogastric tube. Control drainage to the screw hole through a separate incision in the right hypochondrium. The surgical wound is sutured in layers. Postoperative diagnosis: Acute pancreatitis. Jejunum diverticulosis complicated by diverticulitis.

Fibrogastroduodenoscopy on 30.10.25. The esophagus is passable. His mucous membrane is pink. The cardia socket closes. The gastric mucosa is loose, edematous, and there are areas of erosion up to 0.3 cm in size in the area of the transition to the duodenum. There are no defects in other departments. Conclusion: Chronic gastritis. Erosive duodenitis. Gastroesophageal reflux disease 1st.

Ultrasound examination of the hepatoduodenal zone dated 30.10.25. Condition after laparotomy. Echographic picture of hepatomegaly, diffuse changes in liver parenchyma, pancreas, and chronic cholecystitis.

Infusion, detoxification, antibacterial, analgesic, and anti-ulcer therapy were continued in the surgical department. The postoperative period was smooth. The control drains from the abdominal cavity were removed on the 3rd day.

07.11.25—the skin sutures were removed, the postoperative wound was healed by primary tension.

**Figure 2:**
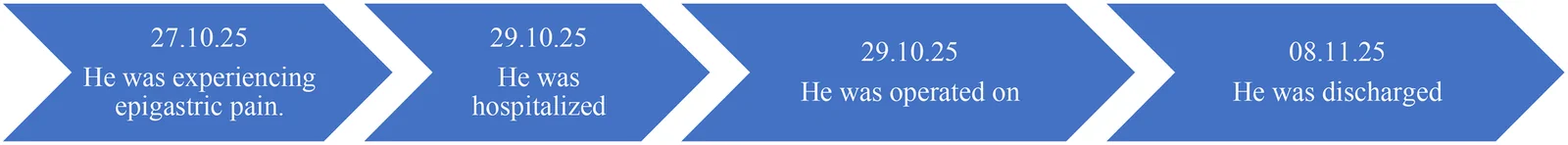
Chronological diagram.

08.11.25—the patient was discharged in satisfactory condition for outpatient treatment. The outcome is recovery. The chronology is shown in Figure 2.

The patient's clinical course demonstrates the complexity of diagnosis and management of jejunal diverticulosis and serves as the basis for the following discussion.

Given the clinical presentation of generalized peritoneal irritation, severe abdominal pain, leukocytosis, and the preoperative diagnosis of a covered perforated gastric ulcer, the patient was considered to require emergency surgical intervention. Contrast-enhanced computed tomography was not performed because it would have delayed definitive treatment in a hemodynamically vulnerable patient with suspected perforation of a hollow viscus requiring urgent exploration. Diagnostic laparoscopy was also not selected because the patient presented with signs of diffuse peritonitis and a high probability of complex intra-abdominal pathology requiring definitive surgical management. Therefore, an emergency exploratory laparotomy was considered the most appropriate approach, allowing complete examination of the abdominal cavity, exclusion of gastric or duodenal perforation, identification of the unexpected jejunal diverticulosis with diverticulitis, and appropriate intraoperative management.

## Discussion

The presented clinical case demonstrates a rare form of jejunal diverticulosis whose clinical manifestations mimicked another acute surgical disease of the abdominal cavity. According to modern data, diverticula of the small intestine may remain asymptomatic for many years, and their first manifestation is frequently associated with the development of complications requiring emergency hospitalization (6, 7). Approximately 10%–30% of patients develop complications such as diverticulitis, intestinal obstruction, hemorrhage, abscess formation, or perforation, making timely diagnosis particularly challenging. Due to the nonspecific clinical picture, the disease is rarely diagnosed before surgery (8).

The etiology of jejunal diverticulosis remains incompletely understood. Unlike Meckel's diverticulum, jejunal diverticula are acquired pseudodiverticula that develop through herniation of the mucosa and submucosa at sites where mesenteric vessels penetrate the muscular layer of the bowel wall. Current evidence suggests that abnormalities of intestinal motility, visceral myopathy, visceral neuropathy, and age-related degeneration of the smooth muscle contribute to increased intraluminal pressure and progressive weakening of the intestinal wall. The predominance of the disease in elderly individuals supports the concept that degenerative changes in the neuromuscular apparatus of the small intestine play a major role in its pathogenesis (1, 7, 14).

One of the most important diagnostic features of the present case was the discrepancy between the severe clinical presentation and the findings of the initial imaging studies. Although the patient had signs of an acute abdomen and diffuse peritoneal irritation, abdominal radiography did not reveal pneumoperitoneum. However, the absence of free subdiaphragmatic air does not exclude perforation of a hollow viscus or other severe intra-abdominal pathology. Previous studies have shown that free intraperitoneal air may be absent in patients with small or temporarily sealed perforations, localized inflammatory processes, or non-perforated jejunal diverticulitis. Therefore, plain abdominal radiography has limited sensitivity for excluding urgent surgical diseases and should always be interpreted in conjunction with the clinical findings (7, 15).

In the presented case, the leading clinical manifestations were pain, intestinal paresis, and signs of acute abdomen, which initially suggested a perforated gastric ulcer. Similar diagnostic difficulties have been described by other authors, who reported that jejunal diverticulitis may mimic intestinal obstruction, acute pancreatitis, perforated peptic ulcer, toxic megacolon, and other emergency abdominal conditions (14, 16, 17).

Several imaging modalities are available for diagnosing small bowel diverticulosis and its complications, although their diagnostic performance varies considerably. Plain abdominal radiography is usually the first imaging examination in patients with an acute abdomen because it is rapid and widely available; however, its sensitivity for detecting small bowel perforation is relatively low, ranging from approximately 50% to 70%, particularly in cases of contained perforation or localized peritonitis. Ultrasonography may demonstrate bowel wall thickening, inflamed diverticula, or localized fluid collections, but its diagnostic accuracy is highly operator-dependent and limited by bowel gas (11).

Contrast-enhanced computed tomography is currently considered the diagnostic modality of choice for suspected jejunal diverticulitis. Recent studies have reported a sensitivity of approximately 80%–95% and a specificity exceeding 90% for identifying diverticulitis and its complications, including abscess formation, contained perforation, bowel wall thickening, inflammatory fat stranding, and extraluminal air. Nevertheless, despite its high diagnostic accuracy, CT findings may remain nonspecific, and the definitive diagnosis is still established intraoperatively in a substantial proportion of patients because of the rarity of the disease and its nonspecific clinical presentation (10, 11, 15).

The choice of surgical access also deserves particular attention (18). Although minimally invasive surgery has become increasingly common in emergency abdominal surgery, exploratory laparotomy remains the preferred approach in patients with diffuse peritoneal irritation and a high suspicion of perforation requiring rapid exploration of the abdominal cavity. In the present case, emergency laparotomy provided complete inspection of the stomach, duodenum, small intestine, and other abdominal organs, allowing exclusion of the suspected perforated peptic ulcer and identification of multiple inflamed jejunal diverticula as the true source of the patient's symptoms. Furthermore, open exploration enabled reliable assessment of bowel viability, exclusion of transmural necrosis and perforation, and selection of the most appropriate surgical procedure.

Currently, most researchers believe that surgical management should be determined not by the presence of diverticula *per se* but by the nature and severity of complications. Resection of the affected small bowel segment is generally recommended in cases of free perforation, bowel necrosis, uncontrolled hemorrhage, diffuse fecal peritonitis, or extensive inflammatory destruction of the intestinal wall (15, 19–21).

However, bowel-preserving surgery may be justified when inflammation is limited and intestinal viability is maintained. Recent publications emphasize that unnecessary bowel resection should be avoided whenever possible, particularly in elderly patients or in those with multiple diverticula, because extensive small bowel resection increases the risks of anastomotic leakage, postoperative morbidity, adhesive bowel obstruction, and, in extensive resections, short bowel syndrome (15, 19, 21).

In our case, despite the presence of multiple diverticula and signs of diverticulitis, there were no signs of perforation or irreversible destruction of the intestinal wall, which allowed us to avoid intestinal resection and limit the procedure to sanitation and drainage of the abdominal cavity. The intraoperative assessment demonstrated preserved bowel viability without ischemia or transmural necrosis, supporting an organ-preserving surgical approach. A favorable postoperative outcome confirms that conservative surgical management may be appropriate in carefully selected patients without bowel perforation or irreversible intestinal damage (19, 20).

The issue of diagnosis deserves special attention. Despite the widespread introduction of computed tomography, accurate preoperative diagnosis of small bowel diverticulosis remains difficult. According to contemporary reviews, even with modern imaging techniques, the final diagnosis is frequently established only during emergency surgical exploration because of the rarity of the disease and its nonspecific clinical manifestations (6, 15).

Our case demonstrates that careful intraoperative assessment remains the cornerstone of decision-making in complicated jejunal diverticulosis. The extent of surgery should be based on bowel viability, the presence or absence of perforation, ischemia, necrosis, and the degree of intra-abdominal contamination rather than on the mere presence of diverticula. This individualized approach allows preservation of healthy bowel whenever it is clinically safe while ensuring adequate control of the inflammatory process (15, 18, 19, 21, 22).

Thus, diverticulosis of the small intestine should be considered in the differential diagnosis of intestinal obstruction and other acute surgical conditions. Importantly, the absence of pneumoperitoneum on plain abdominal radiography should not exclude this diagnosis when clinical findings indicate an acute abdomen requiring emergency surgery. Likewise, although computed tomography is the preferred imaging modality for suspected small bowel diverticulitis, immediate operative intervention may be justified in patients with convincing signs of generalized peritonitis. In the absence of perforation and irreversible changes in the intestinal wall, diverticulosis itself is not an absolute indication for small bowel resection. The choice of surgical intervention should be individualized according to the severity of complications and the intraoperative findings (15, 19–22).

## Conclusion

Jejunal diverticulosis is an uncommon disorder that may present as an acute surgical abdomen and closely mimic more common emergency conditions, making preoperative diagnosis particularly challenging. The present case demonstrates that the absence of pneumoperitoneum on plain abdominal radiography does not exclude clinically significant intra-abdominal pathology requiring emergency surgical exploration. Although contrast-enhanced computed tomography is currently the preferred imaging modality for suspected jejunal diverticulitis, immediate operative intervention remains justified in patients with convincing signs of generalized peritonitis or suspected perforation of a hollow viscus. Careful intraoperative assessment is essential for establishing the correct diagnosis and determining the optimal surgical strategy. In the absence of bowel perforation, ischemia, or irreversible intestinal damage, bowel-preserving management without intestinal resection may be a safe and effective treatment option. This case emphasizes the importance of individualized surgical decision-making based on intraoperative findings rather than on the mere presence of jejunal diverticula.

## Data Availability

The original contributions presented in the study are included in the article/Supplementary Material, further inquiries can be directed to the corresponding author/s.
